# Computationally efficient modeling of proprioceptive signals in the upper limb for prostheses: a simulation study

**DOI:** 10.3389/fnins.2014.00181

**Published:** 2014-06-25

**Authors:** Ian Williams, Timothy G. Constandinou

**Affiliations:** ^1^Department of Electrical and Electronic Engineering, Imperial College LondonLondon, UK; ^2^Center for Bio-Inspired Technology, Institute of Biomedical Engineering, Imperial College LondonLondon, UK

**Keywords:** proprioceptive feedback, neuroprosthesis, neuromusculoskeletal model, upper limb, biomechanics, muscle spindles, golgi tendon organ, static optimization

## Abstract

Accurate models of proprioceptive neural patterns could 1 day play an important role in the creation of an intuitive proprioceptive neural prosthesis for amputees. This paper looks at combining efficient implementations of biomechanical and proprioceptor models in order to generate signals that mimic human muscular proprioceptive patterns for future experimental work in prosthesis feedback. A neuro-musculoskeletal model of the upper limb with 7 degrees of freedom and 17 muscles is presented and generates real time estimates of muscle spindle and Golgi Tendon Organ neural firing patterns. Unlike previous neuro-musculoskeletal models, muscle activation and excitation levels are unknowns in this application and an inverse dynamics tool (static optimization) is integrated to estimate these variables. A proprioceptive prosthesis will need to be portable and this is incompatible with the computationally demanding nature of standard biomechanical and proprioceptor modeling. This paper uses and proposes a number of approximations and optimizations to make real time operation on portable hardware feasible. Finally technical obstacles to mimicking natural feedback for an intuitive proprioceptive prosthesis, as well as issues and limitations with existing models, are identified and discussed.

## 1. Introduction

A device capable of giving an amputee a sense of feeling back from their prosthetic limb could help millions of people live happier, more productive lives (Blank et al., 2010; Weber et al., 2012). Graded sensory feedback of almost any sort could feasibly provide the user with proprioceptive information about their prosthesis, and haptic, visual, auditory, vibratory and electrocutaneous feedback have all been explored (Clippinger et al., 1974; Ohnishi et al., 2007). However, sensory substitution methods such as these, partially deprive the user of another of their senses and suffer from long training periods and high cognitive load as they require the user to learn and interpret the information encoded by the feedback stimuli. Despite decades of experimentation, sensory substitution has not seen significant clinical application (Ohnishi et al., 2007) and it is an approach that is likely to be increasingly difficult to implement in future as prosthesis complexity increases and the quantity of feedback increases correspondingly.

Direct neural feedback in the form of a neural prosthesis has the potential to provide high quality and intuitive feedback. The incredible capability of neural prostheses to transform lives has already been vividly demonstrated in recent years by the rise of cochlear implants for the deaf and the tantalizing progress in retinal implants for the blind. A proprioceptive prosthesis on the other hand could in theory provide a user with feedback of their limb's position, motion and the forces it is exerting, as well as potentially providing therapeutic benefit for phantom limb issues (Dhillon and Horch, 2005) and a number of groups worldwide are working on developing just such a device (Dhillon and Horch, 2005; Hsiao et al., 2011; Weber et al., 2012; Williams and Constandinou, 2013b).

The ideal for a sensory neural prosthesis would be to mimic naturally occurring neural patterns and stimulate the appropriate neurons with those patterns—providing the user with comprehensive feedback that is as intuitive as possible. However, major obstacles remain to be overcome (see section 4.1), and it seems likely that neural prostheses will rely on the brain's ability to interpret limited and abnormal feedback for some time yet.

Mimicking the function and signals of specialized neurons is an active area of focus for cochlear and retinal prostheses in order to enhance the user's ability to interpret the feedback. The brain's ability to adapt and learn is impressive, but fitting in with its pre-existing neural processing may offer better performance. However, this progression, (from simple graded stimulation to systems of modulation that mimic natural patterns) has not yet been addressed for proprioception, despite tantalizing indications that limited but appropriate neural stimulation can generate limb state representations in the brain (Weber et al., 2011).

**Figure 1:**
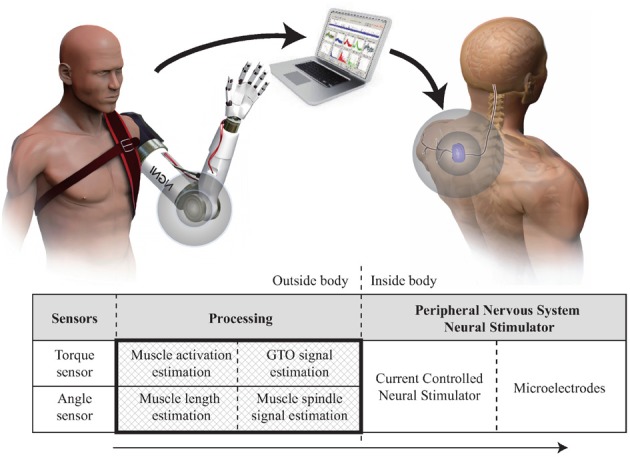
**Proprioceptive prosthesis concept**. Crosshatched area indicates the part of the system presented here.

The aim of this paper is to create a real time model of proprioceptive signals from specific receptors to demonstrate its feasibility and to support future work investigating the possible benefits of mimicking natural signals. Our concept for developing a proprioceptive prosthesis for a transhumeral amputee is shown in Figure 1 and involves mapping the motion of a prosthetic onto a model of the human arm so that equivalent representations of muscle, tendon and receptor modulation can be calculated. Section 4.1 discusses this approach and looks at some of the issues involved. This paper will focus on the processing element—creating an efficient model to convert data from sensors on a prosthetic limb into estimates of proprioceptive neural signals from muscle spindles and Golgi Tendon Organs (GTOs).

In the human body the generation of proprioceptive neural signals is implicitly linked with musculoskeletal biomechanics as well as muscle and proprioceptor dynamics (Proske and Gandevia, 2012). The neural signal generation in our proprioceptive prosthesis is likewise based on these three factors and shares much in common with neuro-musculoskeletal models developed for research in the field of human motor control (Lan et al., 2005; Frigon and Rossignol, 2006; Koo and Mak, 2006; Song et al., 2008; Colacino et al., 2010).

The integration of sensory feedback models with representations of musculoskeletal components is still a relatively new field and most publications have focused on the lower limb and locomotion. Upper limb models considering only 1 degree of freedom have previously been proposed (Lan et al., 2005; Koo and Mak, 2006; Colacino et al., 2010) and a more complex 3 degree of freedom, 15 muscle “Virtual Arm” model covering shoulder and elbow joints was proposed by Song et al in Song et al. (2008). These studies focus on understanding limb motion and control and therefore simplifications and qualitative representations of proprioceptive signals are used which are unlikely to be suitable for implementation in a proprioceptive prosthesis due to limitations that include: using models fitted to feline firing patterns despite much lower observed firing rates in humans; using individual receptor or ensemble firing patterns interchangeably even though there may be multiple orders of magnitude difference between the two; and using simple piecewise linear approximations to population firing rates that do not capture the observed dynamics or non-linearities of proprioceptive receptors. These models also have muscle activation or excitation as inputs, and limb movement as the output. However, in a proprioceptive prosthesis the situation is reversed with limb movement as an input and muscle activation an unknown. Therefore, despite the similarities in the underlying sub-models, the final model implemented here differs substantially.

The addition of static optimization (an inverse dynamics tool) is proposed to estimate muscle forces and activations. However, standard implementations are computationally demanding—unsuited to the real-time, portable, and low-power nature of a proprioceptive prosthesis—and as such approximations are proposed to address this.

Numerous models of muscle spindles have been proposed in the literature [see Prochazka and Gorassini (1998) and Mileusnic et al. (2006a) for review]. Here the anatomically derived Mileusnic et al muscle spindle model (Mileusnic et al., 2006a) will be used and adjusted to fit human spindle firing rates for a variety of muscles in the upper limb. The model is relatively computationally intensive and as such an approximation to this model will be proposed to reduce the computational load.

There are relatively fewer GTO models in the literature [see Mileusnic et al. (2006b) for review]; here the model described in Lin and Crago (2002) (based on a transfer function model by Houk and Simon) was selected for implementation. A method to fit this model to human data and adjust the model according to optimal isometric muscle strength is proposed.

This paper proposes a system for modeling ensemble average proprioceptor signals (see section 4.1 for discussion of this approach) for a simplified representation of the upper limb with 7 degrees of freedom and 17 muscles. Approximations to existing models and tools are proposed with the aim of creating a real time system capable of running on portable hardware.

## 2. Models and methods

**Figure 2:**
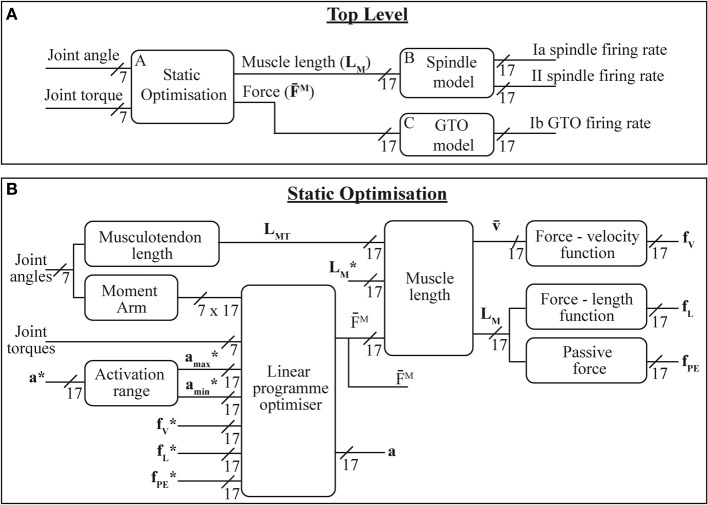
The models presented here: (A) overall system model; (B) static optimization model—(^*^) indicate state variable values from previous iteration. Variable labels are explained in section 2.1.3.

The system described here is shown in Figure 2 and broadly consists of biomechanical modeling combined with two previously described proprioceptor models. The sensor data from the prosthetic limb consists of joint angles and torques mapped onto the joints of the modeled human limb; the role of the biomechanical modeling is to convert this data into estimates of muscle length and force. These parameters are in turn converted by the receptor models into estimates of neural firing patterns.

### 2.1. Musculoskeletal model

#### 2.1.1. Skeletal structure

**Figure 3:**
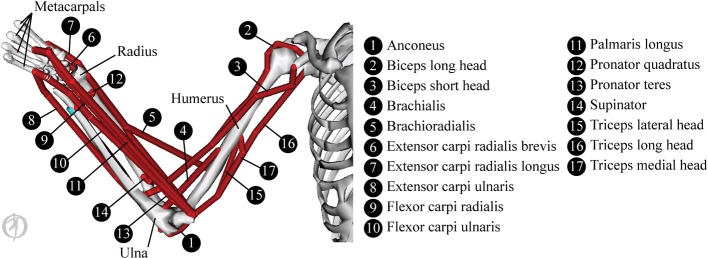
**The musculoskeletal model, showing the paths of the 17 muscles modeled here**.

The biomechanical modeling is underpinned by data from a 3D musculoskeletal model of the upper limb in OpenSim (Delp et al., 2007). The OpenSim model used here is a reduced form of the Stanford VA Upper Limb model which is based on the measurements and proposals in Holzbaur et al. (2005). The reduced form of the model is shown in Figure 3 and consists of the following skeletal elements: thorax, sternum, scapula, clavicle, humerus, radius, ulna, wrist bones and 2nd to 5th metacarpals. Mass and inertial properties were obtained from Chandler et al. (1975) and Winter (2009) and the mass and inertia of the not-included finger and thumb segments were approximated as a lumped mass at the center of gravity of the hand.

#### 2.1.2. Joints and degrees of freedom

The model covers 7 degrees of freedom in the upper limb: 3 at the shoulder (describing elevation angle, shoulder elevation and shoulder rotation), 2 at the elbow (covering elbow flexion and forearm pronation), and 2 at the wrist (covering flexion and deviation). Joint kinematics and ranges of motion were unchanged from the original model.

#### 2.1.3. Muscle model

A standard three component dimensionless Hill type muscle model (0° pennation angle) was used and scaled to fit individual muscles as proposed by Zajac (1988). This approach allows all muscles to be modeled by the same functions with the differences between each muscle described by only a few variables. Normalized muscle length ($L^{\begin{array}{l} -\\ M \end{array}}$), tendon length ($L^{\begin{array}{l} -\\ T \end{array}}$), muscle force ($F^{\begin{array}{l} -\\ M \end{array}}$) and muscle velocity ($v^{\begin{array}{l} -\\ M \end{array}}$), were respectively calculated using:

(1) $$L^{\begin{array}{l} -\\ M \end{array}}=\frac{L^{M}}{L_{o}^{M}},\;L^{\begin{array}{l} -\\ T \end{array}}=\frac{L^{T}}{L_{s}^{T}},\;F^{\begin{array}{l} -\\ M \end{array}}=\frac{F^{M}}{F_{o}^{M}},\;v^{\begin{array}{l} -\\ M \end{array}}=\frac{v^{\;M}}{v_{max}^{M}}$$

where *L^M^_o_* is the optimal muscle length, *L^T^_s_* is the tendon slack length, *F^M^_o_* is the muscle's maximum isometric force and *v^M^_max_* is the muscle's maximum shortening velocity. All muscle paths and muscle insertion points are as specified in the OpenSim model. Parameters for the muscles were obtained from the OpenSim model and *v^M^_max_* was assumed to be seven times the optimal fiber length [a figure approximately midway between that recorded for slow and fast twitch fibers (Brooks and Faulkner, 1988; Thelen, 2003)].

The torque (*T*) produced by the 17 muscles (*m*) around joint (*j*) is modeled as:

(2) $$\begin{array}{l} T_{j}=\sum_{m=1}^{17}([a_{m}\;\cdot \;f_{l}(L_{m}^{\begin{array}{l} -\\ M \end{array}})\;\cdot \;f_{v}(v_{m}^{\begin{array}{l} -\\ M \end{array}})\\ \;+f_{p}(L_{m}^{\begin{array}{l} -\\ M \end{array}})]R_{m,j}\;\cdot \;F_{o,m}^{M}) \end{array}$$

where *f_l_*, *f_v_*, and *f_p_* are functions describing the muscle's force-length, force-velocity, and passive force-length relationships; *a_m_* is the level of muscle activation (between 0 and 1); *R_m,j_* is the muscle's moment arm around joint *j*; and *F^M^_o,m_* is muscle *m*'s maximum isometric force. Equations for calculating force-velocity, force-length, and passive force are as described by Thelen (2003).

### 2.2. Biomechanical modeling

#### 2.2.1. Musculotendon length and muscle moment arms

In OpenSim the musculotendon length and muscle moment arms are calculated based on the muscle's origin and insertion points as well as anatomical wrapping points and constraints. However, running a 3D model is computationally intensive. A more efficient (although less accurate) approach based on fitting a polynomial surface to the length-joint angles relationship and another for the moment arm-joint angles relationship was described in van den Bogert et al. (2011).

In order to determine the polynomial coefficients for this relationship, the OpenSim musculoskeletal model was swept through the full range of motion of the various joints and at each pose the lengths and moment arms of all the muscles were recorded. This data was then processed in Matlab with the polyfitn function to generate polynomial surfaces fitted to this data. The polyfitn function outputs the polynomial surface coefficients (*c^L^_i_* for length and *c^MA^_i_* for moment arm) relating the musculotendon lengths (*L*^*MT*^) and muscle moment arms (*R_m,j_*) to the joint angles (*q_j_*) for muscle *m* such that:

(3) $$L_{m}^{MT}=\sum_{i\;=\;1}^{N^{L}}c_{i}^{\;L}\prod_{j\;=\;1}^{7}q_{j}^{e_{\theta }^{L}},\;R_{m,j}=\sum_{i\;=\;1}^{N^{MA}}c_{i}^{MA}\prod_{j\;=\;1}^{7}q_{j}^{e_{\theta }^{MA}}$$

where *N^L^* and *N*^*MA*^ are the number of polynomial terms for length and moment arm respectively, while *e*^*L*^_θ_ and *e*^*MA*^_θ_ are the integer exponents for length and moment arm respectively. A cubic polynomial fit was used, therefore giving *e*^*L*^_θ_ and *e*^*MA*^_θ_ values between 0 and 3.

The length of the muscle (*L^M^*) was calculated from the musculotendon length, by subtracting an estimate of the tendon length under tension. This estimate of tendon length was based on the recorded strain curve of normalized tendons as described in Thelen (2003). For efficient modeling this force strain relationship was approximated by computing the equilibrium position (removing differential equations) using a cubic polynomial fitted to the strain curve:

(4) $$L^{\begin{array}{l} -\\ T \end{array}}=0.04879F^{\begin{array}{l} -\\ M \end{array}}{}^{{}^{3}}-0.1009F^{\begin{array}{l} -\\ M \end{array}}{}^{{}^{2}}+0.1003F^{\begin{array}{l} -\\ M \end{array}}+1$$

#### 2.2.2. Muscle activations and forces

It has been widely noted that there is redundancy in the human musculoskeletal system and hence there is typically not a unique combination of muscle forces to generate any particular motion. The situation is further complicated by the fact that muscles are often multi-articular and produce moments around each of the joints they span. Methodologies such as static or dynamic optimization [which rely on minimizing or maximizing some optimization criteria (Erdemir et al., 2007)] are often used to address this redundancy and complexity problem. Due to the real time nature of this system, the static optimization technique will be used here.

Probably the simplest proposed optimization criteria is to try to minimize the total amount of muscle activation ($\sum_{m\;=\;1}^{17}a_{m}$). This approach has the advantage of being linear and hence solvable by fast linear programme solvers, however, this optimization approach does not produce results that are representative of observed patterns of muscle activation (Rasmussen et al., 2001). There is still no clear agreement on the best optimization criteria for all joints, motions and loads, however, representative muscle activations have been produced by systems minimizing sum of activation squared, cubed or to a higher order polynomial ($\sum_{m\;=\;1}^{17}a_{m}^{n}$ where *n* is an integer greater than 1). However, solving these criteria requires significantly higher computational power than the linear criteria. In Rasmussen et al. (2001), Rasmussen proposed using a min-max optimization criteria which approximates the high order polynomial, but which can be solved using efficient linear techniques. This optimization criteria can be formulated by introducing an artificial criterion variable (β):

Minimize β subject to:

(5) $$\begin{array}{l} a_{m}\leq \beta ,\;\forall m\in \{1,2,\ldots 17\}\\ 0\leq a_{m}\leq 1\\ T_{j}^{*}=\sum_{m\;=\;1}^{17}\left(\left[a_{m}\;\cdot \;f_{l}({\bar{L}}_{m}^{M})\;\cdot \;f_{v}({\bar{v}}_{m}^{M})\;+\;f_{p}({\bar{L}}_{m}^{M})\right]R_{m,j}\;\cdot \;F_{o,m}^{M}\right),\\ \forall j\in \{4,\;5,\;6,7\} \end{array}$$

where, T^*^_j_ is the measured torque around joint *j* and the muscle activations (*a_m_*) are the variables for the algorithm.

A weakness of this optimization criteria is that once a minimum β value has been calculated, the optimization process does not try to reduce muscle activations below this value, e.g., if muscle *i* needs to be fully activated (*a_i_* = 1, β = 1) there is no optimization penalty for setting other muscles to be fully activated. To address this the optimization criteria was modified, with the new aim being to minimize $\beta +0.01\sum_{m\;=\;1}^{17}a_{m}$.

The open source simplex package lp_solve was used to solve this linear programme around the four joints in the elbow and wrist. Given the limited subset of shoulder spanning muscles being modeled (and the target application being a transhumeral amputee with extant shoulder musculature), it was not possible to resolve the torques at the three shoulder joints (*j* = 1, 2, 3). These joints were, however, included in the system because their configuration influences the lengths of and moments developed by the bicep and tricep muscle groups around the elbow and hence have an impact on all distal muscle activations.

#### 2.2.3. Activation dynamics

Muscle activation dynamics were accounted for by using a method similar to that used by Thelen (2003). In that paper an idealized muscle excitation signal (*u*) was used as an input and the muscle activation (*a*) was modeled by a non-linear first order differential equation:

(6) $$\frac{da}{dt}=\frac{u-a}{\tau_{a}(a,u)}$$

where τ_*a*_ is a time constant that varies depending on the muscle activation level and on whether the activation level is increasing or decreasing:

(7) $$\tau_{a}=\{\begin{array}{ll} \tau_{act}(0.5+1.5a) & \;u>a\\ \tau_{deact}/(0.5+1.5a) & \;u\leq a \end{array}$$

where τ_*act*_ is 15 ms and τ_*deact*_ is 50 ms.

In our work we do not have the muscle excitation (*u*) available so the problem was addressed by determining the feasible range of activation levels (*a*_*min*_ → *a_max_*) each muscle could have after a time *dt*. This was approximated from the differential equation describing the muscle activation by setting *u* = 0 to determine *a*_*min*_ and *u* = 1 to determine *a*_*max*_, giving:

(8) $$\begin{array}{l} a_{min}=a-\frac{dt\cdot a\cdot (0.5+1.5a)}{\tau_{deact}}\\ a_{max}=a+\frac{dt(1-a)}{\tau_{act}(0.5+1.5a)} \end{array}$$

The feasible activation range was calculated for each muscle and included as constraints in the linear programme solver.

### 2.3. Proprioceptor modeling

#### 2.3.1. Muscle spindles

The muscle spindle outputs were simulated using a model based on that proposed by Mileusnic et al. (2006a). The model inputs are muscle length (*L^M^*) and fusimotor activation levels (γ_*static*_ and γ_*dynamic*_). The model uses this to estimate the tension in each spindle fiber type's (*bag*_1_, *bag*_2_, and *chain*) transduction zone, and the resulting action potential firing. The output of the model is a non-linearly summed contribution from each fiber type to the primary (Ia) and secondary (II) axons that innervate the spindle. The model works essentially by modeling the fibers as a spring mass system and solving a second order differential equation (Equation (6) in Mileusnic et al., 2006a) that describes the tension in each fiber. However, integration of this differential equation requires a small time step size and therefore a high number of calculations. We propose that the tension in the system can be approximated by assuming that all the stretch happens in the polar regions of the fiber (which have a much lower spring constant) and then calculating the equilibrium tension in the fibers by modifying Equation (3) in Mileusnic et al. (2006a) to:

(9) $$\begin{array}{l} T=M\cdot \overset{..}{L^{M}}+\beta \cdot C\cdot (L^{M}-R-L_{o}^{sr})\cdot (abs{\left(\dot{L^{M}}\right)}^{0.3})\\ \;\cdot sign(\dot{L^{M}})+K^{pr}\cdot (L^{M}-L_{o}^{pr}-L_{o}^{sr})+\Gamma \end{array}$$

where *T* is the fiber tension, *L^M^* is the muscle length, *C* is the coefficient of asymmetry in the muscle force-velocity curve, *R* is the muscle length below which force production is zero, *L^pr^_o_* and *L^sr^_o_* are the rest lengths of the polar and sensory parts of the fiber, *K*^*pr*^ is the polar region spring constant and Γ is the tension produced due to fusimotor input. This modification means that there are no differential equations to solve, so the time step for calculating the spindle output can be increased by orders of magnitude and the computational efficiency is likewise improved.

The parameter “G” in the Mileusnic model is a scaling term—mapping ideal normalized spindle firing rates to feline data in the paper—and was estimated based on changes in spindle firing rates of up to 150 pulses per second (pps), that occur due to fusimotor stimulation in a feline muscle. There is limited data about the fusimotor sensitivity of human muscle spindle, but the maximum observed change in spindle output due to fusimotor signals has been observed to be <30 pps (Prochazka and Hulliger, 1998) and as such we scaled the Mileusnic et al. (2006a) derived values of “G” by a factor of $\frac{1}{5}$ to better fit human spindle firing rates.

#### 2.3.2. Golgi tendon organs

The GTO model used here is based on the model described by Lin and Crago (2002), which in turn is based on work by Houk and Simon (1967) studying the feline soleus muscle. The model consists of two stages.

Firstly a non-linearity:

(10) $$R^{NL}=k_{1}\cdot ln\text{ ​}\left(\frac{F^{M}}{k_{2}}+1\right)$$

where *R*^*NL*^ is the output of this stage, while *k*_1_ (60 impulses per second) and *k*_2_ (4 Newtons) are constants scaling the GTO firing rate to the force applied. However, these parameters are based on data from the feline soleus muscle, and given the limited amount of data from human recordings it is difficult to determine human appropriate values for these parameters. We propose to modify this non-linearity to use normalized muscle force:

(11) $$R^{NL}=k_{1}\cdot ln\text{ ​}\left(F^{\begin{array}{l} -\\ M \end{array}}\cdot \frac{F_{o,s}^{M}}{k_{2}}+1\right)=k_{1}\cdot ln\;(F^{\begin{array}{l} -\\ M \end{array}}\cdot k_{3}+1)$$

where *F^M^_o,s_* is the maximum isometric muscle force of the feline soleus [measured as 25.8N (Scott et al., 1996)], giving *k*_3_ a value of 6.45. In addition we propose to adjust *k*_1_ to reflect the lower observed firing rates in human GTOs compared to feline GTOs (Jami, 1992). Feline GTOs have been observed firing at rates of up to 300 pps, but in normal motion don't significantly exceed 120 pps (Jami, 1992). A review of the literature did not find any examples of human GTOs being subject to tests that would produce maximal firing rates, however, a review of microneurographic recordings showed firing patterns that rarely exceed 50 pps in normal motions (al Falahe et al., 1990; Jami, 1992). We therefore propose a *k*_1_ figure scaled accordingly of 25 pps.

Secondly, the output of the non-linearity is then fed into a linear dynamics transfer function:

(12) $$H(s)=\frac{1.7s^{2}+2.58s+0.4}{s^{2}+2.2s+0.4}.$$

For efficient implementation this transfer function was transformed into the z-domain in Matlab using a bilinear approximation with a sample frequency of 1 kHz and warped to fit at 6 Hz giving a z-domain transfer function of:

(13) $$H(z)=\frac{1.69942-3.39626z^{-1}+1.69684z^{-2}}{1-1.99780z^{-1}+0.99780z^{-2}}.$$

## 3. Results

### 3.1. Model validation

The focus of the work presented here is on choosing and modifying existing validated models to create a real time system. As such the approximations will be validated against the original models and the computational efficiency compared. To generate a dataset for realistic comparison, 30 s (at 120 Hz) of motion capture data from the mocapdata.com website (product_id = 15,044 showing an actor swinging his arms while walking across a room, then brushing his teeth before walking back to the original spot) was scaled, fed into OpenSim and the resulting joint angles for the upper limb were used for all simulations. This data set was chosen because it has a range of fast and slow upper limb motions and because tooth brushing represents an example of where a prosthesis user would not be able to visually monitor their limb and so feedback could provide significant benefit.

#### 3.1.1. Length and moment arm validation

The polynomial approximation for estimating length showed close conformance with the values generated by OpenSim's 3D model throughout the dataset; giving a coefficient of determination (*R*^2^) of in excess of 0.99 for all muscles. The fit of the moment arm approximation was slightly worse with *R*^2^ values of in excess of 0.9 for the two bicep muscles and above 0.98 for all other muscles for the four joints of interest.

#### 3.1.2. Static optimization validation

**Figure 4:**
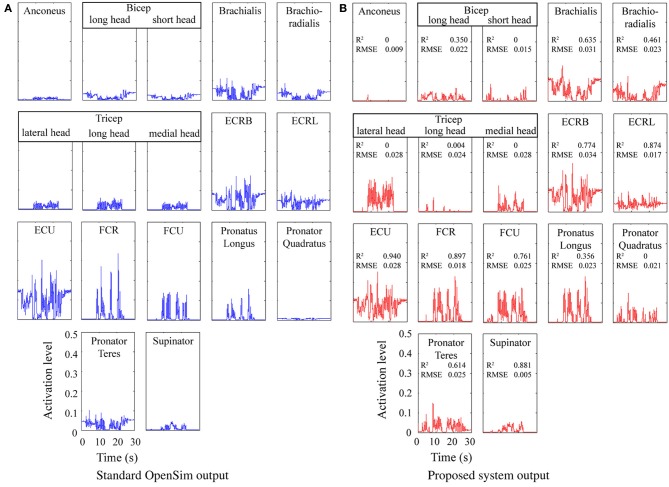
**Predicted muscle activations for an actor brushing his teeth**. Coefficient of determination (R^2^) and Root Mean Square Error (RMSE) are shown for each. **(A)** Standard OpenSim output. **(B)** Proposed system output.

Figure 4 shows a comparison between a baseline static optimization tool and results obtained from the model proposed here. The baseline results were obtained by running the built-in OpenSim static optimization tool using a sum of activation squared optimization criteria. Muscle forces and moment arms from OpenSim were used to estimate the joint torques at each point in time and these torques were used as the inputs to the model described here. The results indicate the proposed system can produce results qualitatively very similar to existing standard techniques, however, it did flag up an issue in the load sharing between the tricep muscles (see Discussion).

#### 3.1.3. Spindle model validation

**Figure 5:**
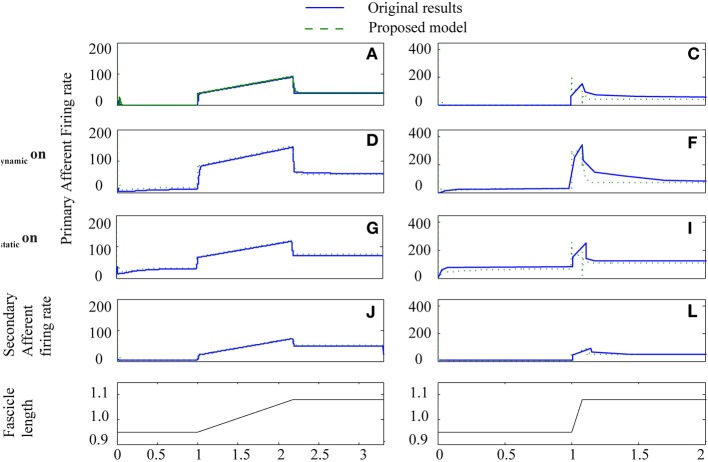
**Proposed spindle model compared to original results from paper Mileusnic et al. (2006a) for two triangular stretches with different levels of fusimotor activity**. Results are from Figure 3: **A–L** showing primary and secondary afferent firing in the presence or absence of static or dynamic fusimotor activation at 70 pps. Labels are as in original paper.

**Figure 6:**
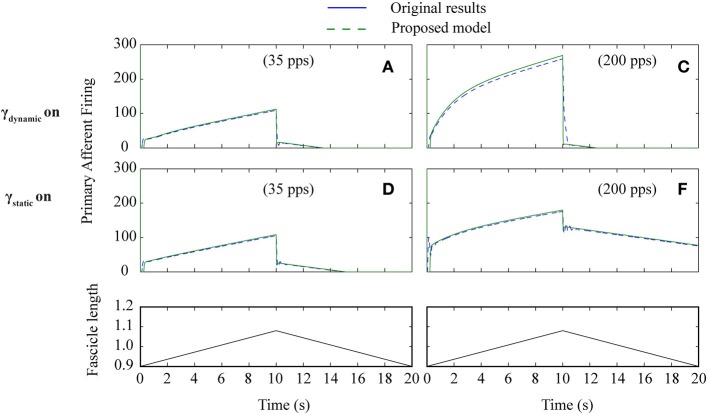
**Proposed spindle model compared to original results (Figure 4) from paper Mileusnic et al. (2006a) for triangular stretches with different levels of fusimotor activity**. Results are from Figure 4: **A–F** showing primary afferent firing in the presence of static or dynamic fusimotor activation at 35 or 200 pps. Labels are as in original paper.

**Figure 7:**
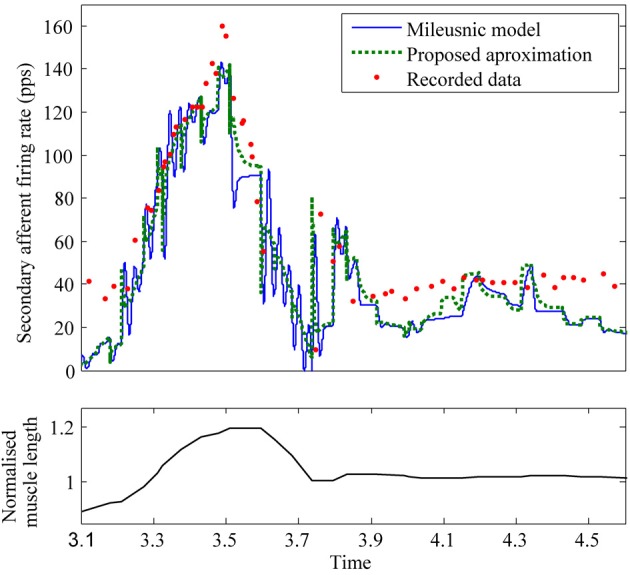
**Comparison of Mileusnic modeled data and proposed approximation against recorded data from a cat (Figure 4A of Prochazka et al., 1979)**. Fusimotor activity was assumed to be absent.

To test how well the equilibrium spindle approximation corresponded to the original Mileusnic et al model, a number of the validation runs from the original paper were repeated and the results are shown in Figures 5, 6. Differences are generally small and this is consistent with the observed performance for more complex data (as shown in Figure 7), although the proposed spindle model is stiffer and as a result rapid movements do give rise to minor discrepancies—the most notable of which is shown in Figure 5F.

**Figure 8:**
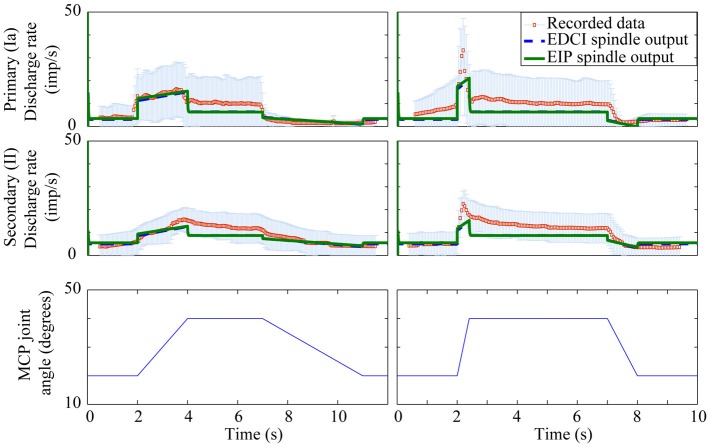
**Human muscle spindle firing patterns for imposed motions**. Mean values and one standard deviation error bars are shown for recorded data from Edin and Vallbo (1990) and contrasted with modeled primary and secondary spindle firing patterns for the EDCI and EIP muscles. Firing patterns for EDCI and EIP muscles are almost identical. Fusimotor input was assumed to be absent.

There is little data available to assess the models performance for human spindles, however, the limited validation possible did highlight a potential limitation of the Mileusnic model which is discussed below. Figure 8 shows a comparison with the recordings, from two sets of nine primary afferents and two sets of seven secondary afferents, published in Edin and Vallbo (1990). The recordings are from the radial nerve during imposed motions about the metacarpophalangeal (MCP) joint. Predicted firing patterns were generated by using the original Holzbaur et al model in OpenSim to estimate the lengths of the two extensor muscles innervated by the radial nerve [extensor digitorum communis interossei (EDCI) and extensor indicis proprius (EIP)] during MCP joint motions as described in the paper.

Spindle lengths vary significantly less from muscle to muscle than muscle fasicle lengths do, this discrepancy is possible because the spindle attachment to muscle endpoints or perimysium is varied to provide consistent proprioceptive acuity across a range of joints and muscles (Proske et al., 2000). The Mileusnic et al model was optimized for muscles whose fiber length varies above and below the optimal fiber length, whereas both the EDCI and EIP muscles are physiologically constrained to be longer than their optimal lengths. As such the unmodified model overestimates spindle firing rates throughout the range of motion (even in the absence of fusimotor input). Spindle rest and threshold lengths in the model were therefore adjusted to correspond to the fiber length when the MCP joint is in a physiologically neutral position (0° flexion) rather than the optimal muscle length. This approach gives the results shown in Figure 8.

#### 3.1.4. Full system output

**Figure 9:**
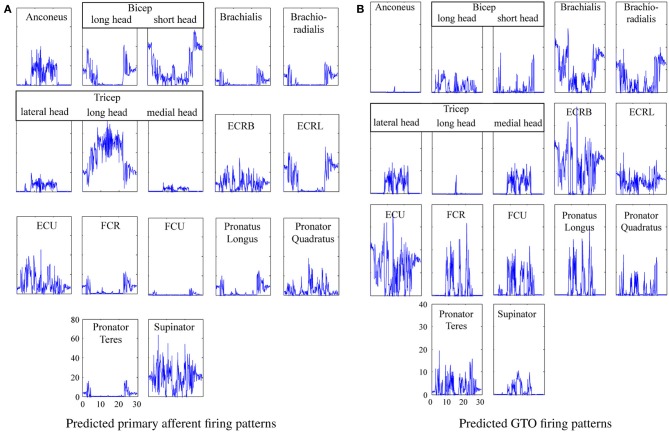
**Predicted proprioceptor firing patterns for the actor brushing his teeth. (A)** Predicted primary afferent firing patterns. **(B)** Predicted GTO firing patterns.

The system was run to generate primary afferent and GTO neural signals to enable 3rd party validation of this work, and the neural firing patterns are shown in Figure 9.

### 3.2. Computational efficiency

For the purposes of this analysis the code was broken down into two parts—a deterministic part and a non-deterministic part. The deterministic part consisting of the newly written code (calculating muscle lengths, moment arms, force-velocity/length relationships, muscle spindle output, etc.), while the non-deterministic part of the code (formulating and solving the optimization linear programme) used an open source library.

The 30 s 120 Hz dataset (consisting of 3600 samples) was processed by a 2.1 GHz laptop in under 1.09 s (i.e., 27.5 times faster than real time). Profiling showed that 15% of that time was spent in the deterministic part of the code and 85% in the non-deterministic optimization code. A manual estimate of the number of instructions required to process each time sample (based on the source code) was conducted for the deterministic part of the code—yielding a value of approximately 77,000 instructions. A conservative estimate of the total number of instructions (deterministic and non-deterministic) necessary for processing each sample was made based on scaling the estimated number of instructions by the relative processing duration of the deterministic and non-deterministic parts and then multiplying by a factor of 2—this provided an estimate of 1.03 million instructions per sample.

#### 3.2.1. Comparison with standard implementations

Comparisons between the proposed spindle model and a C-code implementation of the standard Mileusnic model (using a standard euler method solver) showed that the majority of the improvement in processing speed was due to differences in the time steps that can be used (rather than reduction in the number of calculations per time step). The standard Mileusnic model can become unstable if too large a time step is chosen (experimentation showed that a maximum timestep in the order of 0.1–1 ms is required, depending on the dataset), whereas the proposed solution is stable regardless of the timestep. As such it was necessary to upsample the 120 Hz dataset used here, by a factor of 8, to obtain results with the standard model but not for the proposed model. However, the maximum timestep for the proposed model will be upper bounded by the limb position update frequency and the maximum firing frequency of the spindle—meaning that the efficiency improvement is data dependent—but in this situation was in the region of an eightfold improvement.

The fitting of cubic polynomials to calculate length and moment arm make these elements of the processing almost negligible and appears to represent a reduction in required calculations by multiple orders of magnitude compared to 3D modeling. Observed execution time for all the biomechanical modeling (length, moment arm and also the static optimization) presented here was around five orders of magnitude faster than in OpenSim, however, this is a deeply unfair comparison—pitting optimized C-code against the performance of the general purpose OpenSim package.

## 4. Discussion

### 4.1. Generating proprioceptive feedback for a prosthesis

Providing intuitive and comprehensive feedback which is familiar or trivially easy to interpret is the ultimate goal for any neural prosthesis. However, it is unknown what the most effective and achievable format for providing proprioceptive neural feedback to prosthesis users is in the near term and there are numerous challenges in implementing, comparing and optimizing competing methods.

Stimulating a small number of neurons with a pattern that is linearly related to prosthetic limb parameters (e.g., joint angle or end effector force) is possibly the simplest approach and has been demonstrated to provide benefit in a laboratory control task (Clippinger et al., 1974; Dhillon et al., 2004; Dhillon and Horch, 2005; Dhillon et al., 2005; Rossini et al., 2010). Stimulation of non-proprioceptive neurons with these non-biologically representative patterns makes this approach similar to sensory substitution feedback. It is unknown whether sensory substitution feedback using direct neural stimulation will offer any substantial benefit over non-implanted implementations although the ability to stimulate truncated nerves and neural pathways that would otherwise be silent may confer advantages.

An alternative feedback modulation method is one that aims to approximate all the naturally occurring neural feedback patterns in the human limb. This approach is in its infancy and there is a need for physiological experimentation to verify the suitability of this approach and investigate how close to this ideal the feedback needs to be in order to demonstrate benefit compared to simpler forms of modulation, however, it seems a logical, albeit distant, ideal to aim for. Major obstacles remain such as limitations in our understanding of proprioceptors and the modulating signals from the brain, as well as our limited ability to interface with and selectively modulate large numbers of neurons. Our concept for implementing this approach breaks the process down into three steps:

1. **Mapping from a prosthesis to a model of a normal limb**
  Prosthetic limb properties can differ substantially from those of a human limb. For the purposes of this mapping the differences between the human and prosthesis can be grouped into three main categories: (a) physical properties—weight, moments of inertia, size and shape (including for instance the number of digits); (b) actuation properties—strength, speed, joint coupling and actuator non-linearities; and (c) kinematic properties—degrees of freedom, range of motion, axis of rotation and joint structure (including for instance joint complexes). These differences were most evident in the days when cable and hook prostheses dominated the market. However, under the twin pressures of prosthesis users' desire for cosmesis and functionality (in a world of tools and equipment designed to be operated by the human hand), there has been a strong trend toward anthropomorphic convergence. Prototype upper limbs such as the DEKA arm or commercially available prostheses such as the i-Limb, clearly demonstrate the progress that has been made toward approximating the human upper limb. Even the rise in underactuation for finger joints—which was largely driven by actuator weight considerations—moves prosthetics closer to the human form and with anthropomorphic design as a guiding principle, it is a trend that looks set to continue.
  This has important implications because the closer the match between prosthetic limbs and human limbs, the easier the process of mapping the state of one to the other becomes.
2. **Modeling proprioceptor behavior**
  Assuming a modeled human limb can match the states and motion of the prosthesis, then modeling the neural signals can be consdered a problem of biomechanics and proprioceptor modeling (assuming feedback is applied in the Peripheral Nervous System).
  Receptors in the muscles, joints and skin all sense tissue deformation and provide proprioceptive feedback to the CNS. Ideally all these receptors would be modeled for a proprioceptive prosthesis so that appropriate stimulation could be applied to any receptors interfaced with. However, in a system constrained by power, portability and (as a result) complexity, it is necessary to prioritize. Discriminating criteria include the importance of each receptor type to motor control, our ability to model the underlying tissue deformation and our understanding of the receptor firing patterns.
  Here we elected to focus on muscle spindles and GTOs, both of which stand out on the grounds of the quality of information they provide to the CNS and the quality of the models available for musculo-tendon and proprioception modeling. However, even for these relatively well understood receptors modeling limitations are evident such as a lack of parameters for human receptors, difficulties fitting parameterized models to different muscles and uncertainty regarding fusimotor input (see section 4.3 for further discussion).
3. **Applying appropriate neuromodulation to enough target neurons**
  When electrodes are implanted in or around a nerve, it is unknown which neurons will be stimulated and what sensation or motor effect they can elicit. The main factors in determining which neurons are stimulated are the electrode-neuron distance, the stimulus strength and the neuronal diameter (with larger neurons recruited at lower thresholds). There is typically a trade-off between the number of neurons stimulated and the selectivity achieved; with stimulation of non-target neurons with a synchronous barrage leading to unusual or potentially noxious sensations (Smith and Leslie, 1990). Possible techniques to reduce the number of non-proprioceptive neurons stimulated include: careful choice of implantation nerve or nerve branch to increase the ratio of proprioceptive neurons stimulated (versus motor or exteroceptive neurons); electrodes with designs that increase selectivity and provide greater ability to target stimulation at different fascicles or at the sub-fascicular level; and waveforms that alter the distance and diameter recruitment order.
  The stimulation pattern to apply potentially depends on how selectively individual neurons can be targeted. Our approach is to target fascicle level selectivity because higher selectivity electrodes typically need to penetrate the perineurium which introduces a break in the blood-nerve barrier and has been observed to cause endoneurial accumulation, fibrous build up due to tissue rejection and neural damage caused by relative motion (boring at the electrode tip) (Biran et al., 2005; Polikov et al., 2005). At the fasicular level we propose to stimulate with ensemble average signals—with the aim of making use of the ability of the central nervous system to integrate feedback. The extent to which the CNS can interpret a subset of normal feedback (even in the presence of contradictory feedback) is largely unquantified, but is demonstrated by single muscle tendon vibration trials and numerous psychophysical experiments over the years examining proprioceptive performance under varying conditions including local anesthesia.

### 4.2. Approximations

The system presented here is focused on real time prediction of neural stimulation patterns and firing rates that would be suitable for human nerve stimulation and which are based on data that could be available from a prosthetic limb. A number of models and approximations were used to achieve this aim. The cubic polynomial approximations for muscle length curves fitted the OpenSim data closely, however, the equivalent moment arm approximations showed substantially worse correlation. It was observed that the quality of a muscle's moment arm approximation decreased as the number of joints the muscle spanned increased and that the surface fit for moment arms about the shoulder joints were particularly poor (although that did not matter for this application). Considering these differences in input data and the difference in optimization criteria employed, the output of the static optimization stage showed a reasonably good match with the standard OpenSim tool. However, substantial differences were visually evident in the distribution of load between the three tricep muscle branches and this is reflected in the very low R^2^ values for these branches. This was likely due to the linear nature of the optimization, which becomes increasingly poor at load sharing as the number of joints and muscles increases. Examination of the results showed that simply averaging the activations of the three tricep branches would have closely fitted the OpenSim results (R^2^ values of 0.912, 0.970, and 0.906 for the lateral, long and medial heads respectively). In more complex systems (with greater numbers of joints and muscles) it may be necessary to compartmentalize the optimization process and run multiple iterations or implement some alternative method of sharing load. Results for bicep long and short head activation were also well below average and may be a result of the poorer moment arm fit observed for these muscle branches.

The simplified version of the spindle model closely matched the outputs of the original model for the validations proposed in the original paper as well as for some real movement data. Discrepancies were visible during rapid movements, however, given the duration of these transient differences and peak firing rates in humans of approximately 100 Hz, these discrepancies represent only a low number of missed action potentials. As mentioned in the results, the efficiency improvement provided by the proposed model appears to be largely data dependent and related to the sample frequency of the system, the maximum spindle output frequency and the maximum step size for stable solving of the differential equations in the standard model. It should be noted that the analysis here assumed a standard euler method for solving these equations, but that many alternative numerical methods exist and could improve or guarantee stability.

The proposed parameter change and adjustment to rest and threshold lengths allowed the model to estimate human spindle recordings to within a standard deviation, but without a significantly greater quantity of human data it is unclear how widely applicable these adjustments are.

### 4.3. Issues and areas for further work

- The proposed approach of mimicking naturally occurring neural signals seems logical, however, physiological experimental to demonstrate and quantify benefit remains an important area of future work.
- The inaccuracy of the polynomial fitting of moment arms for shoulder and highly multi-articular muscles indicates that alternative fitting models may need to be investigated to achieve good fits if the system is to be extended to include other joints.
- The scaling of animal spindle and GTO models to fit human data is an area requiring further investigation. Here we have proposed simple modifications to better align the models with human firing rates, however, further data, validation and modification is required. The shape of the modeled human firing patterns was qualitatively different from the recorded data—most notably in that it lacked an initial burst or peak. Further analysis of recorded human spindle data from Cordo et al. (2002) (not shown), has supported this observation and indicates that the Mileusnic spindle model does not accurately represent the human spindle firing profile observed. Initial peaks are generated with the Mileusnic spindle model, but only at significantly faster movements—potentially indicating greater acceleration or velocity sensitivity in human spindles compared to feline or an area requiring further investigation and potentially model modification.
- In addition the Mileusnic spindle model is formulated on a limited subset of muscles and may need modification for wider applicability. In this work we noted a need for further research on how well the model copes with muscles whose optimal length is above or below the range of lengths the muscle is physiologically limited to. In our work looking at the recordings in Edin and Vallbo (1990), we made the assumption that the muscle physiological rest length should be used to calculate spindle rest and threshold parameters, this was based on the assumption that spindles firing rates should be at their lowest when the muscle is in a relaxed and neutral position.
- The work presented here assumed zero fusimotor activity, producing signals that are analogous to those experienced during passive motion of the limb. However, if the user is able to control their prosthesis naturally (i.e., the prosthesis responds to physiologically appropriate neural commands—e.g., following Targeted Muscle Reinnervation), then there will be a descending fusimotor signal that will act in the CNS on the pathways carrying the stimulated proprioceptive feedback, potentially interfering with it and reducing effectiveness. Ultimately it would be preferential to integrate fusimotor behavior and the Mileusnic spindle model was in part chosen to enable future implementations to easily introduce this. However, current proposed models of fusimotor action are in their infancy and largely based on recordings performed on decerebrate cats or surrogate outcomes like obtaining a linear relationship between joint angle and spindle firing (Taylor et al., 2004, 2006; Williams and Constandinou, 2013a).
- The integration of motion capture, a musculoskeletal model, static optimization and proprioceptor models enables some proprioceptive signals to be non-invasively modeled for real movements. Further integration of inverse dynamic modeling to estimate joint torques, as well as a suitable musculoskeletal model of the feline hind limb (a preferred experimentation model), would enhance this system, allowing novel experimentation and extensive validation.

## 5. Conclusion

Realistic models that link human motion to proprioceptor signals could 1 day form the basis for a proprioceptive neural prosthesis in much the same way retinal and cochlear implants aim to mimic auditory and retinal cells. In contrast to previous neuro-musculoskeletal models, this work has proposed: the integration of static optimization; modifications to approximate human proprioceptors; and a variety of approximations and optimizations to reduce computational complexity without substantial degradation of the output. A key uncertainty in aiming to provide natural feeling proprioceptive feedback to a prosthesis user is how close to normal it needs to be in order to provide benefit over simpler forms of feedback modulation. This work aims to build capability to explore this question.

The model presented here is able to simulate muscle lengths, moment arms and activations as well as the corresponding muscle spindle and GTO neural signals in real time on low power hardware. This system potentially enables physiological experimentation into intuitive proprioceptive feedback as well as novel forms of proprioceptive and motor control and maybe 1 day could form part of a system capable of giving amputees feeling in their prosthetic limbs.

### Conflict of interest statement

The authors declare that the research was conducted in the absence of any commercial or financial relationships that could be construed as a potential conflict of interest.
